# Early transcriptional responses to human enteric fever challenge

**DOI:** 10.1128/iai.00108-23

**Published:** 2023-09-19

**Authors:** Amber Barton, Jennifer Hill, Daniel O'Connor, Claire Jones, Elizabeth Jones, Susana Camara, Sonu Shrestha, Celina Jin, Malick M. Gibani, Hazel C. Dobinson, Claire Waddington, Thomas C. Darton, Christoph J. Blohmke, Andrew J. Pollard

**Affiliations:** 1 Oxford Vaccine Group, Department of Paediatrics, University of Oxford and the NIHR Oxford Biomedical Research Centre, Oxford, United Kingdom; 2 Department of Pathology, Royal Melbourne Hospital, Melbourne, Australia; 3 Infectious Diseases and Immune Defence Division, Walter and Eliza Hall Institute of Medical Research, Melbourne, Australia; 4 Department of Infectious Disease, Imperial College, London, United Kingdom; 5 Department of Infection, Immunity and Cardiovascular Disease and The Florey Institute for Host-Pathogen Interactions, University of Sheffield, Sheffield, United Kingdom; University of California San Diego School of Medicine, La Jolla, California, USA

**Keywords:** typhoid, enteric fever, human challenge, *Salmonella*, transcriptomic

## Abstract

Enteric fever, caused by oral infection with typhoidal *Salmonella* serovars, presents as a non-specific febrile illness preceded by an incubation period of 5 days or more. The enteric fever human challenge model provides a unique opportunity to investigate the innate immune response during this incubation period, and how this response is altered by vaccination with the Vi polysaccharide or conjugate vaccine. We find that on the same day as ingestion of typhoidal *Salmonella*, there is already evidence of an immune response, with 199 genes upregulated in the peripheral blood transcriptome 12 hours post-challenge (false discovery rate <0.05). Gene sets relating to neutrophils, monocytes, and innate immunity were over-represented (false discovery rate <0.05). Estimating cell proportions from gene expression data suggested a possible increase in activated monocytes 12 hours post-challenge (*P* = 0.036, paired Wilcoxon signed-rank test). Furthermore, plasma TNF-α rose following exposure (*P* = 0.011, paired Wilcoxon signed-rank test). There were no significant differences in gene expression (false discovery rate <0.05) in the 12 hours response between those who did and did not subsequently develop clinical or blood culture confirmed enteric fever or between vaccination groups. Together, these results demonstrate early perturbation of the peripheral blood transcriptome after enteric fever challenge and provide initial insight into early mechanisms of protection.

## INTRODUCTION

An estimated 14.3 million cases of typhoid and paratyphoid fever, collectively referred to as enteric fever, occur each year (1). Systemic infection is caused by bacterial pathogens *Salmonella enterica* serovars Typhi and Paratyphi A-C, acquired from contaminated food and water. While a protein-polysaccharide conjugate vaccine has been recently demonstrated to be highly effective against typhoid fever in children, no vaccine against paratyphoid fever has yet been licensed (2, 3). *S*. Typhi is thought to invade the intestinal epithelium and disseminate systemically within the first 24 hours after infection (4). Infection is asymptomatic for up to 2 weeks (5), after which a secondary bacteremia occurs, often accompanied by fever and fatigue (6).

It is unclear whether vaccination and natural immunity exert their protective effect on bacterial invasion of the intestinal epithelium at a later stage in infection or at multiple points during infection. Furthermore, mechanisms of protection, which may differ between natural immunity and vaccines, are unclear. The enteric fever human challenge model, where volunteers are orally exposed to typhoidal *Salmonella*, offers a unique opportunity to investigate host-pathogen interactions during the incubation period. Previous studies using the human challenge model have shown that live *S*. Typhi can be detected in stool in the first days after oral challenge, both for participants who go on to develop disease and those who stay healthy (7), suggesting that *S*. Typhi reaches the lower gastrointestinal tract in both groups. Likewise, *S*. Typhi DNA has been detected in the blood in the first few days after the challenge, both in those who later develop typhoid and those who do not (8).

In this study, we apply transcriptomics as an unbiased approach to identifying early host responses to enteric fever, both in unvaccinated and vaccinated participants. Transcriptomics has previously been used to characterize pre-symptomatic host responses to challenge with influenza (9), rhinovirus (10), and malaria (11), providing a broad snapshot of the pathways activated in the first days after exposure. Controlling for the confounding effect of circadian rhythms, we find enrichment of gene sets relating to innate immunity in both those who did and did not go on to develop enteric fever. Furthermore, estimating cell proportions from gene expression data suggested a greater number of monocytes may be in an activated state 12 hours post-challenge relative to baseline. We also find that plasma TNF-α is significantly raised in challenged participants but not unchallenged controls.

## RESULTS

### The blood transcriptome is significantly perturbed 12 hours post-challenge

**Fig 1:**
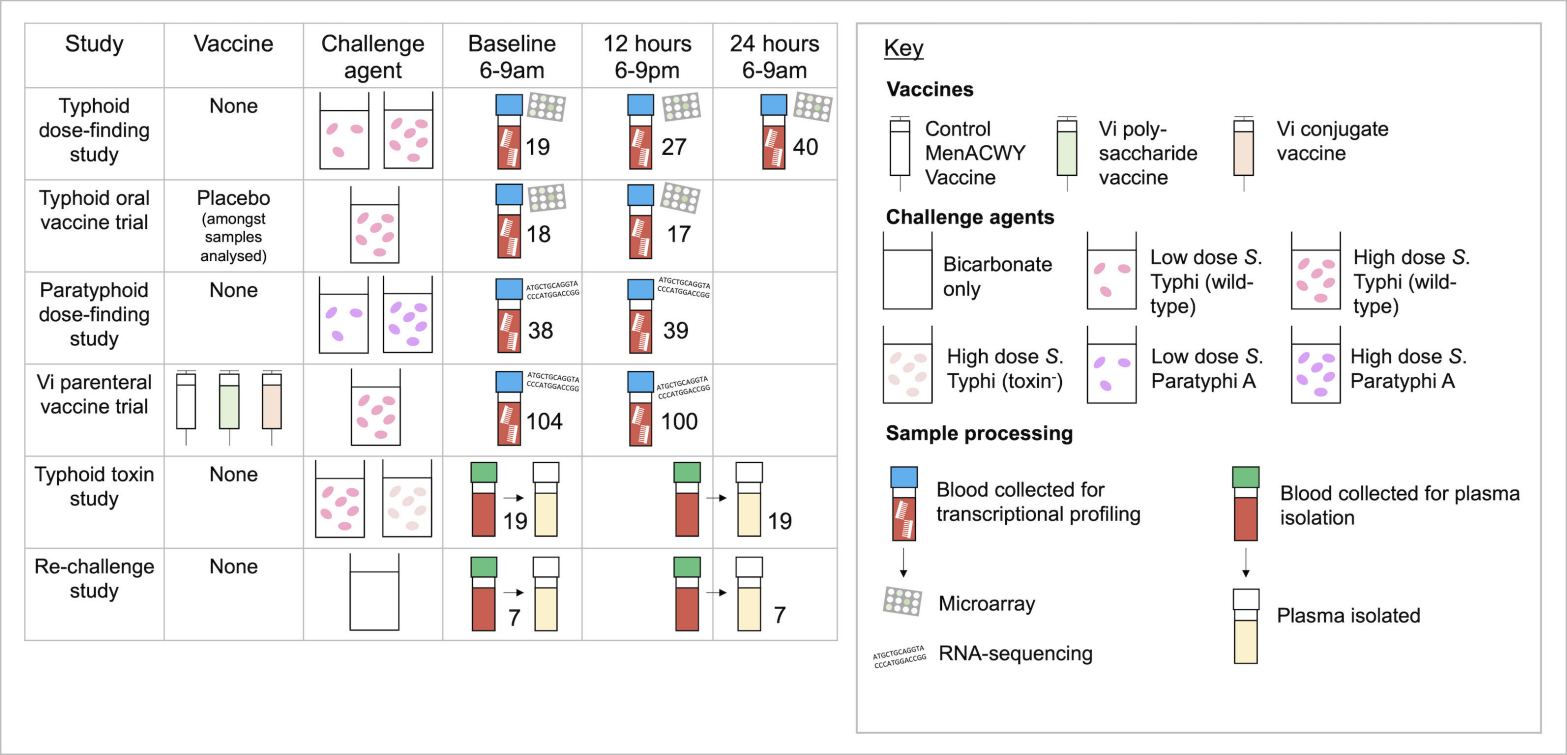
Schematic of the studies and samples analyzed in the following paper. Samples from four enteric fever human challenge studies underwent transcriptional profiling, while plasma samples from two studies were processed and stored for multiplex cytokine assays at the timepoints indicated.

To investigate early responses to human *S*. Typhi and *S*. Paratyphi challenge, whole blood samples from four human challenge studies were transcriptionally profiled (Fig. 1; Table 1). At 12 hours post-challenge relative to baseline in the typhoid dose-finding/typhoid oral vaccine trial data set, Paratyphoid dose-finding study, and typhoid parenteral vaccine trial, 2,611, 456, and 793 genes were differentially expressed (false discovery rate <0.05, linear modeling in limma), respectively. We also re-analyzed two transcriptomic data sets investigating the circadian rhythm in humans (12, 13) in order to identify and exclude genes differentially expressed due to morning collection of baseline samples and evening collection of 12 hours samples. A relaxed definition of a “circadian gene” [unadjusted *P* < 0.05 or absolute log_2_(fold change) >0.1] was chosen to minimize the number of false-positives attributed to enteric fever challenge, particularly as the circadian studies had small sample sizes (32 evening samples in Archer et al., 9 evening samples in Braun et al.). Circadian genes were significantly over-represented (fisher-test, *P* < 0.05) among those differentially expressed following challenge (Fig. S1). Circadian genes from both the Archer et al. and Braun et al. data sets were therefore excluded from the following analyses. This left 859, 193 and 86 differentially expressed genes (false discovery rate <0.05, linear modeling) in the Typhoid dose-finding/Typhoid oral vaccine trial data set, paratyphoid dose-finding study, and typhoid parenteral vaccine trial, respectively (supplementary document 1). In the typhoid dose-finding/typhoid oral vaccine trial data set, challenge dose had no significant effect on gene expression 12 hours post-challenge. However for the paratyphoid dose-finding study, 10 non-circadian genes were significantly different in the 12 hours response between dose groups (false discovery rate <0.05), being upregulated in the higher dose but not the lower dose group (Table S1).

**TABLE 1:** Summary of the Oxford enteric fever human challenge studies and the samples used in this analysis

Study	Groups	Plasma cytokine profiling	Transcriptional profiling
Typhoid dose-finding study (14, 15)	Low dose inoculum (*n* = 20) High dose inoculum (*n* = 20)		Microarray Both low and high dose challenged groups in the typhoid-dose finding study (Baseline *n* = 19, 12 h *n* = 27, 24 h *n* = 40) Unvaccinated group of the typhoid oral vaccine trial (Baseline *n* = 18, 12 h *n* = 17)
Typhoid oral vaccine trial (7)	Ty21a vaccination (*n* = 30) M01ZH09 vaccination (*n* = 31) Placebo (*n* = 30)		
Paratyphoid dose-finding study (16)	Low dose inoculum (*n* = 20) High dose inoculum (*n* = 20)		RNA-sequencing Both groups (Baseline *n* = 38, 12 h *n* = 39)
Typhoid parenteral vaccine trial (17)	Conjugate vaccination (ViTCV) (*n* = 41) Polysaccharide vaccination (ViPS) (*n* = 37) Control MenACWY vaccination (*n* = 34)		RNA-sequencing All groups (Baseline *n* = 104, 12 h *n* = 100)
Typhoid toxin study (18)	Wild-type challenge agent (*n* = 21) Toxin-negative challenge agent (*n* = 19)	Challenged participants Delayed processing of 12 h timepoint (Baseline *n* = 19, 12 h *n* = 19)	
Re-challenge study (19)	Naïve typhoid challenge (*n* = 19) Naïve paratyphoid challenge (*n* = 19) Homologous re-challenge (*n* = 40) Heterologous re-challenge (*n* = 37) Unchallenged controls (*n* = 10)	Unchallenged controls only Delayed processing of 12 h timepoint (Baseline *n* = 7, 12 h *n* = 7)	

**Fig 2:**
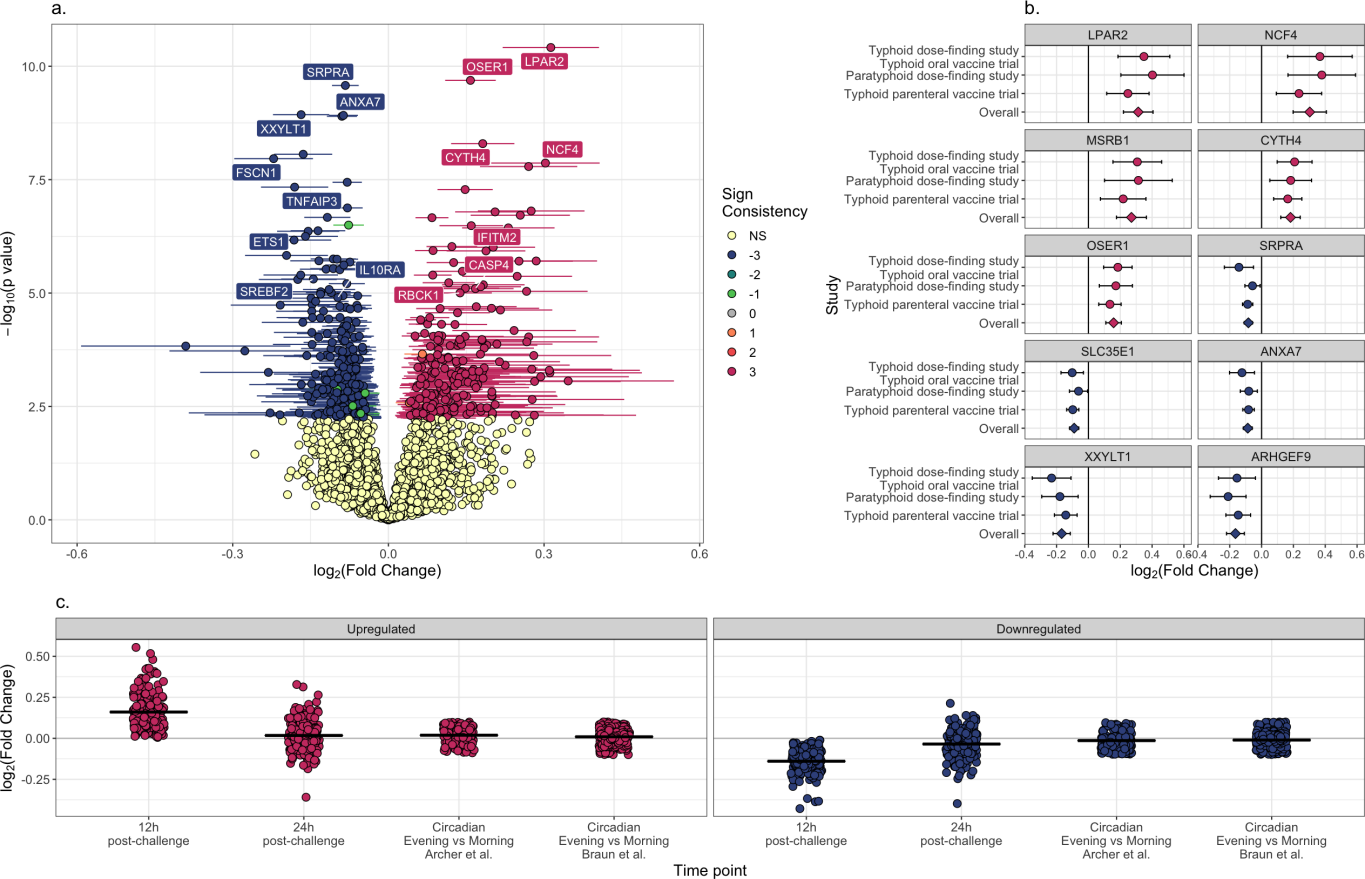
(**a**) Volcano plot [log_2_(fold change) versus –log_10_(unadjusted *P* value)] of genes 12 hours post-challenge relative to baseline in a meta-analysis carried out using MetaVolcanoR. A log_2_(fold change) >0 indicates upregulation. Non-significant genes are colored light yellow, whereas genes with adjusted *P* value < 0.05 are colored by their sign consistency (e.g., a sign consistency of three corresponds to upregulation in all three data sets). Ninety-five percent confidence intervals in log_2_(fold change) for genes with *P* value < 0.05 are indicated by error bars. (**b**) Log_2_(fold change) and 95% confidence intervals for each of the top five most upregulated and downregulated genes for each individual data set and meta-analysis. (**c**) Log_2_(fold change) of genes identified in a meta-analysis as being differentially expressed 12 hours post-challenge, 12 and 24 hours post-challenge in the microarray typhoid dose-finding/typhoid oral vaccine trial data set. The log_2_(fold change) of the same genes in both of the circadian rhythms data sets are shown for comparison. Upregulated genes are colored red and downregulated genes blue. The mean is indicated by a horizontal line.

For the 3,869 non-circadian genes shared among all three data sets, there was a positive correlation in the log_2_(fold change) in gene expression 12 hours post-challenge (Table 2). In order to identify differences in gene expression consistent across all three enteric fever challenge data sets, a meta-analysis using a random effects model approach was carried out (20). *P* values were adjusted for multiple testing using the Benjamini-Hochberg method. Overall, 199 genes were identified as upregulated 12 hours following enteric fever challenge and 219 as downregulated (adjusted *P* value < 0.05 and consistent direction between studies; Fig. 2a and b and supplementary document 2). For three random uncorrelated data sets, we would expect 25% of genes to have a consistent direction of change. Among all 3,869 tested genes, we found that 47% had a consistent direction of change, and among the 427 genes with overall adjusted *P* value < 0.05, 98% had a consistent direction of change. Upregulated genes relating to the innate immune response, as annotated by InnateDB, included *IFITM2*, *CASP4,* and *RBCK1.* Neutrophil cytosolic factor 4 (*NCF4*) was also upregulated and *IL10RA* downregulated. In participants from the typhoid dose-finding study (*n* = 40), the timepoint 24 hours post-challenge was also transcriptionally profiled. By this timepoint, expression of genes which were differentially regulated 12 hours post-challenge had returned to baseline (Fig. 2C).

**TABLE 2:** Spearman correlation coefficient for log_2_(fold change) in genes 12 hours post-challenge relative to baseline compared between data sets

Data set 1	Data set 2	Spearman correlation coefficient
Typhoid dose-finding study/typhoid oral vaccine trial	Paratyphoid dose-finding study	0.44
Typhoid dose-finding study/typhoid oral vaccine trial	Typhoid parenteral vaccine trial	0.23
Paratyphoid dose-finding study	Typhoid parenteral vaccine trial	0.54

### Gene set enrichment analysis and deconvolution suggest innate immune activation

**Fig 3:**
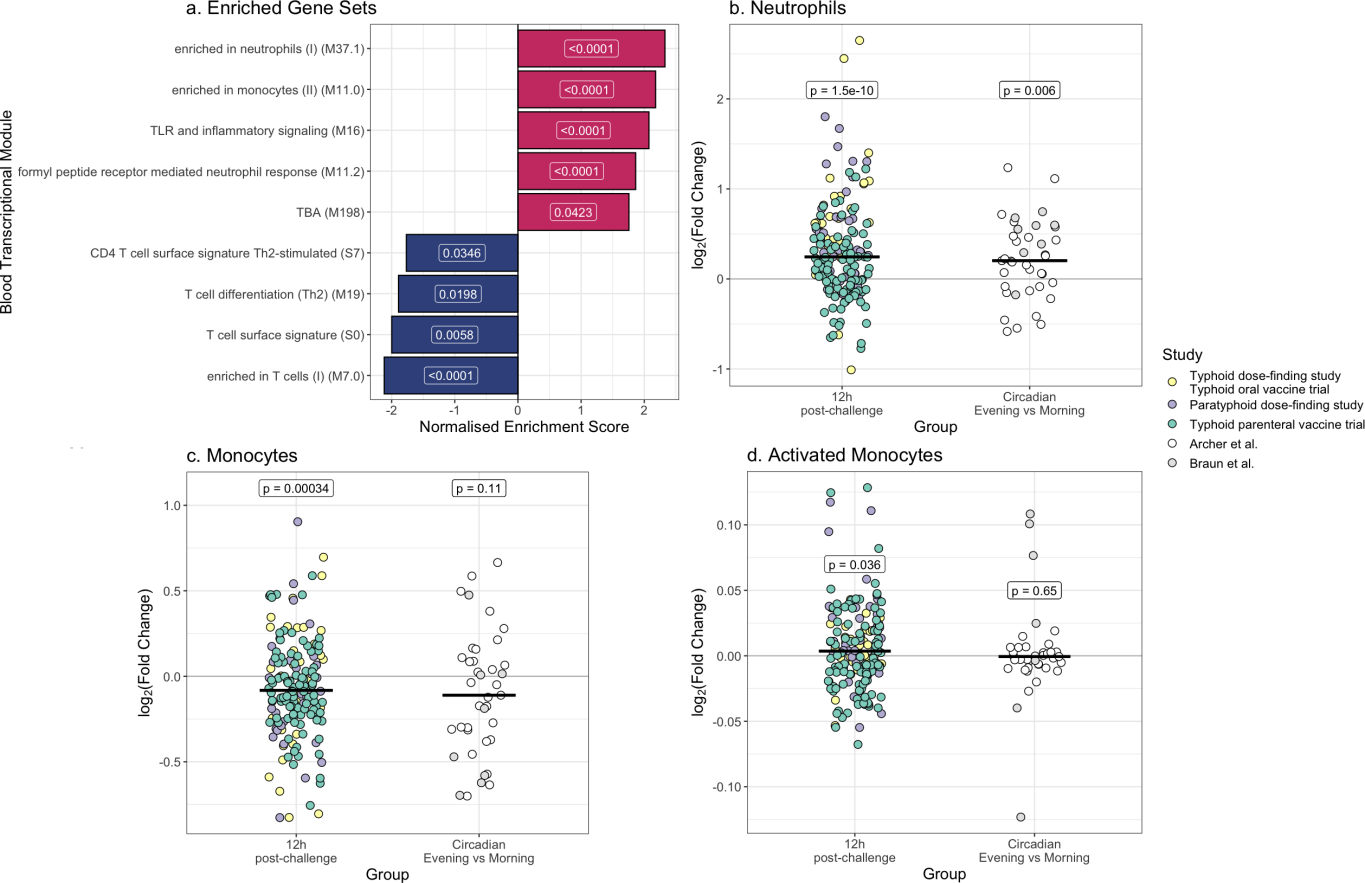
(**a**) Normalized enrichment scores of significantly (adjusted value *P* < 0.05) over- or under-represented blood transcriptional modules at 12 hours post-challenge relative to baseline. Over-represented modules are colored red and under-represented modules blue. Adjusted *P* values are indicated by the labels. (**b**) Log_2_(fold change) in the number of neutrophils estimated by CIBERSORT 12 hours post-challenge relative to baseline. (**c**) Log_2_(fold change) in the number of monocytes estimated by CIBERSORT 12 hours post-challenge relative to baseline. (**d**) Log_2_(fold change) in the number of activated monocytes estimated by the deconvolution algorithm developed by Ben–Moshe et al. Twelve hours post-challenge relative to baseline. Each point represents one participant, colored by study cohort. The log_2_(fold change) in the circadian rhythms data sets are shown for comparison. The median is indicated by a horizontal line. White labels indicate the *P* values for a paired Wilcoxon-signed rank test comparing the 12 hours timepoint with baseline.

Gene set enrichment analysis on the full gene list (3,689 genes following exclusion of circadian genes) ranked by log_2_(fold change) identified enrichment of blood transcriptional modules relating to neutrophils and monocytes and under-representation of gene sets relating to T cells (Fig. 3A). Given the over-representation of blood transcriptional modules relating to innate immune cells 12 hours post-challenge, we next investigated whether this over-representation was likely due to an increased proportion of these immune cells in the blood or increased activation.

Estimated immune cell counts by deconvolution software CIBERSORT significantly correlated with peripheral neutrophil and lymphocyte blood counts measured at baseline (Fig. S2; Pearson’s correlation coefficient *R* > 0.6, *P* < 0.05). Correlation with monocyte blood count was weaker but still positive (*R* = 0.29 and *P* = 0.11 in the typhoid dose-finding study/typhoid oral vaccine trial, *R* = 0.42 and *P* = 0.084 for the paratyphoid dose-finding study; Fig. S2). As whole blood counts were not performed 12 hours post-challenge due to samples being collected in the evening, CIBERSORT was therefore used for *in silico* estimation of changes in immune cell counts following challenge.

Estimated numbers of neutrophils were significantly raised 12 hours post-challenge (*P* = 1.5 × 10^−10^, paired Wilcoxon test). However, combining both circadian data sets neutrophils were raised in the evening (*P* = 0.006, paired Wilcoxon test), suggesting this may be attributable to circadian rhythms. Estimated numbers of monocytes were not raised after challenge, and in fact appeared to decrease (*P* = 0.00034, paired Wilcoxon test). We were, therefore, interested in whether enrichment of blood transcriptional module M11.0 [enriched in monocytes (II)] 12 hours post-challenge was due to monocyte activation rather than increased cell numbers. We applied a dynamic deconvolution algorithm developed by Ben-Moshe et al. (21), which uses a gene signature specifically enriched in *Salmonella* Typhimurium-stimulated monocytes to deconvolute bulk transcriptomics. There was a very small in magnitude, but significant rise in the estimated number of activated monocytes in those who underwent enteric fever challenge (median log fold change of 0.004, *P* = 0.036, paired Wilcoxon test) but not in the circadian comparator data sets (median log fold change of −0.001, *P* = 0.65, paired Wilcoxon test).

### TNF-α is raised in plasma 12 hours post-challenge

**Fig 4:**
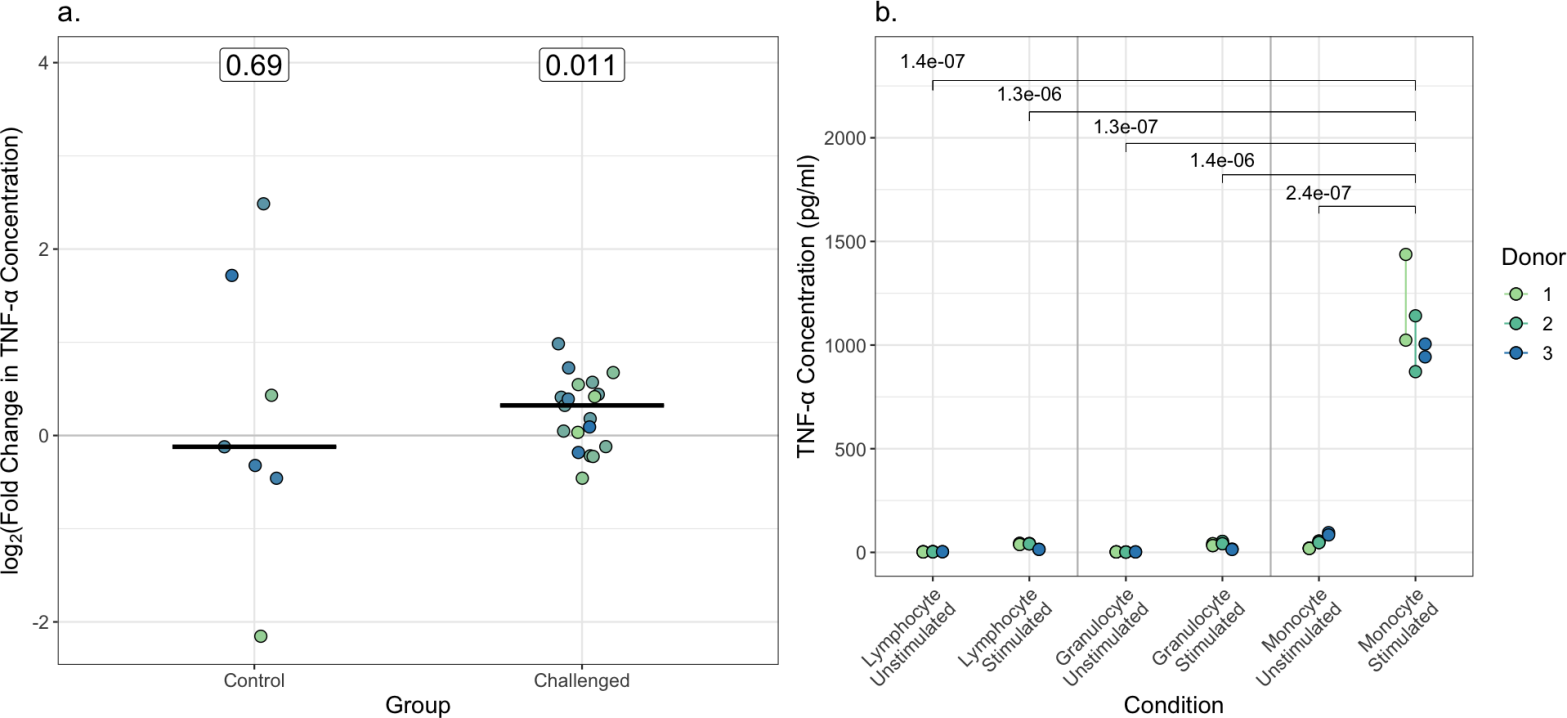
(**a**) The log_2_(fold change) in TNF-α 12 hours after baseline in unchallenged control participants and challenged participants. Each point represents one participant. For each cytokine and group, the median is indicated by a horizontal line. White labels indicate the *P* values for a paired Wilcoxon-signed rank test comparing concentrations at the 12 hours timepoint with baseline. (**b**) TNF-α production by granulocytes, monocytes, and lymphocytes from three donors stimulated with *S*. Typhi at a multiplicity of infection of 10 after 4 hours relative to an unstimulated control. The *P* value for a linear mixed effects model with blood donor modeled as a batch is indicated. Points are colored by donor.

As the predicted number of activated monocytes rose, we were then interested in whether there was any evidence of monocyte activation at the protein level 12 hours post-challenge. A 9-plex cytokine assay, focusing on cytokines previously identified as potentially relevant to the early enteric fever response (14), was performed on plasma samples from the typhoid toxin study (18). We found that TNF-α rises 12 hours following enteric fever challenge (*P* = 0.011, paired Wilcoxon test; Fig. 4a; Fig. S3). As there was a trend toward higher estimated numbers of activated monocytes in the Braun et al. circadian data set, control samples from participants receiving bicarbonate only were also tested. There was no rise in TNF-α in these samples (*P* = 0.69, paired Wilcoxon test) suggesting this change is challenge-specific.

To identify potential sources of TNF-α, the same assay was performed on the supernatants of human monocytes, granulocytes, and lymphocytes stimulated with *S*. Typhi *in vitro* for 4 hours. TNF-α was secreted to a greater extent by activated monocytes than activated granulocytes or lymphocytes (Fig. 4b; Fig. S4; *P* < 0.05, linear mixed effects model with blood donor as a batch).

### There are no early transcriptional differences between those who developed enteric fever and those who remained healthy

**Fig 5:**
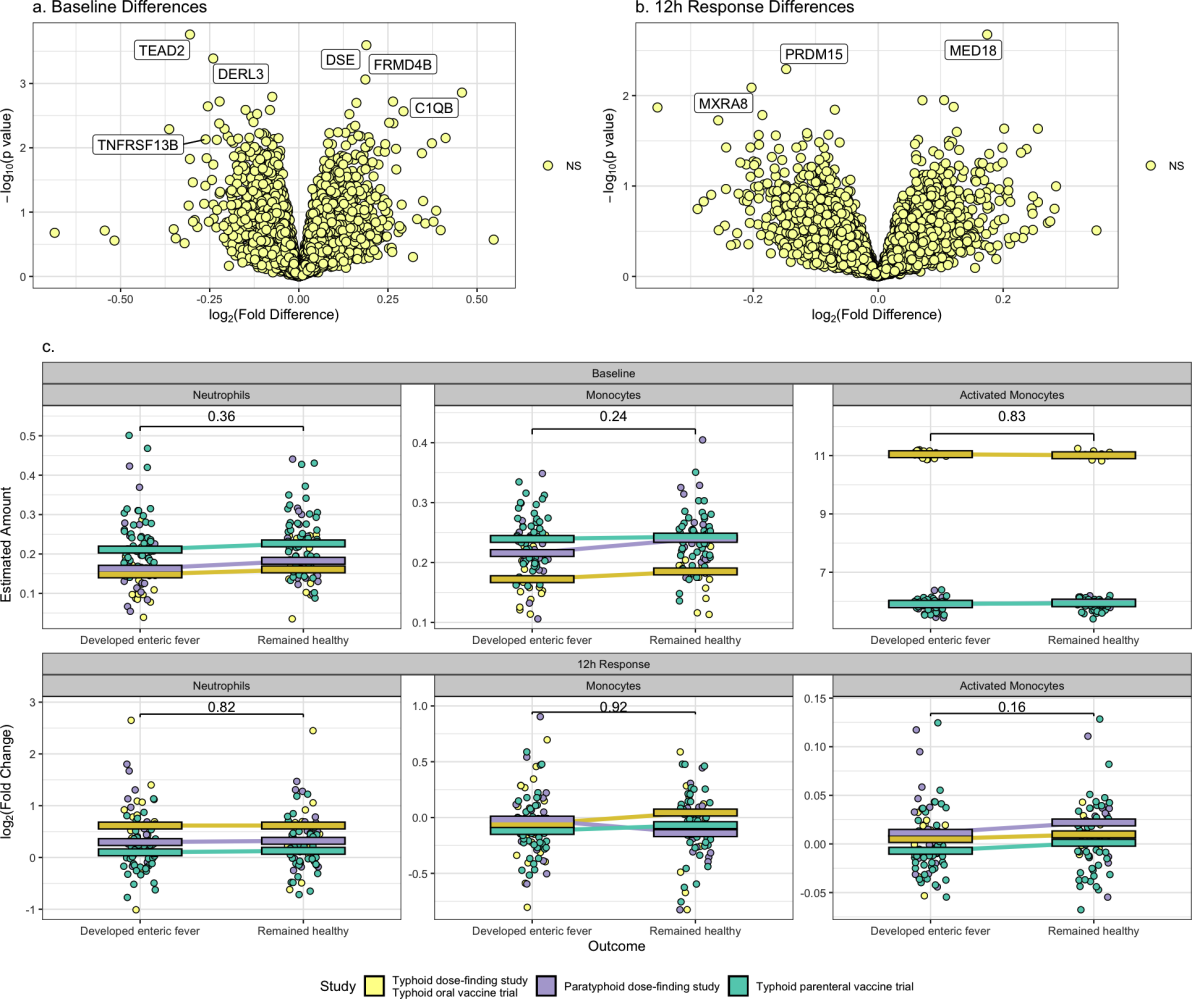
(**a**) Volcano plot [log_2_(fold difference) versus –log_10_(unadjusted *P* value)] of baseline gene expression in those who remained healthy versus those who went on to develop enteric fever in a meta-analysis carried out using MetaVolcanoR. A log_2_(fold difference) >0 indicates a gene having higher expression in those who remained healthy. Non-significant genes after adjusting for multiple testing are colored light yellow. (**b**) Volcano plot [log_2_(fold difference) versus –log_10_(unadjusted *P* value)] in the 12 hours transcriptional response in those who remained healthy versus those who went on to develop enteric fever [i.e., (Remained healthy 12 hours – Remained healthy baseline) – (Developed enteric fever 12 hours – Developed enteric fever baseline)]. A log_2_(fold difference) >0 indicates a greater response in those who remained healthy. Non-significant genes after adjusting for multiple testing are colored light yellow. (**c**) Differences in cell counts estimated by deconvolution between those who remained healthy and those who developed enteric fever. In the top panels, baseline differences in the estimated amount of cells is shown, with a *P* value for a logistic regression adjusting for study as a covariate labeled. In the lower panels, differences in the log_2_(fold change) at 12 hours post-challenge relative to baseline is shown, with a *P* value for a Mann-Whitney test labeled. Each point represents one participant, colored by study. The median for each study is indicated by a colored bar, and the difference in median between the two groups in each study is highlighted by a colored line.

We then investigated whether there were any transcriptional differences between those who went on to develop culture-confirmed or symptomatic enteric fever following challenge and those who did not. In this analysis, genes identified as relating to circadian rhythms or delayed processing were included, as all baseline samples were collected in the morning and processed immediately, and circadian rhythms/delayed processing would have affected 12 hours post-challenge samples from both groups equally. For each data set, we performed differential expression analysis to find whether there were any differences between the two groups at baseline and also whether there were any differences in the 12 hours response. As described above, a random effects model approach was then used to identify differences consistent across all three challenge data sets. After adjusting for multiple testing, there was no differential gene expression at baseline or in the 12 hours response between the two groups (Fig. 5a and b). Nor was there a significant difference in numbers of immune cells estimated by CIBERSORT, although there was a trend toward a greater rise in activated monocytes using the deconvolution algorithm by Ben-Moshe et al. [*P* = 0.16, Mann-Whitney test comparing log_2_(fold change)] in those who remained healthy (Fig. 5c).

### No early differential gene expression was detected between vaccine groups

**Fig 6:**
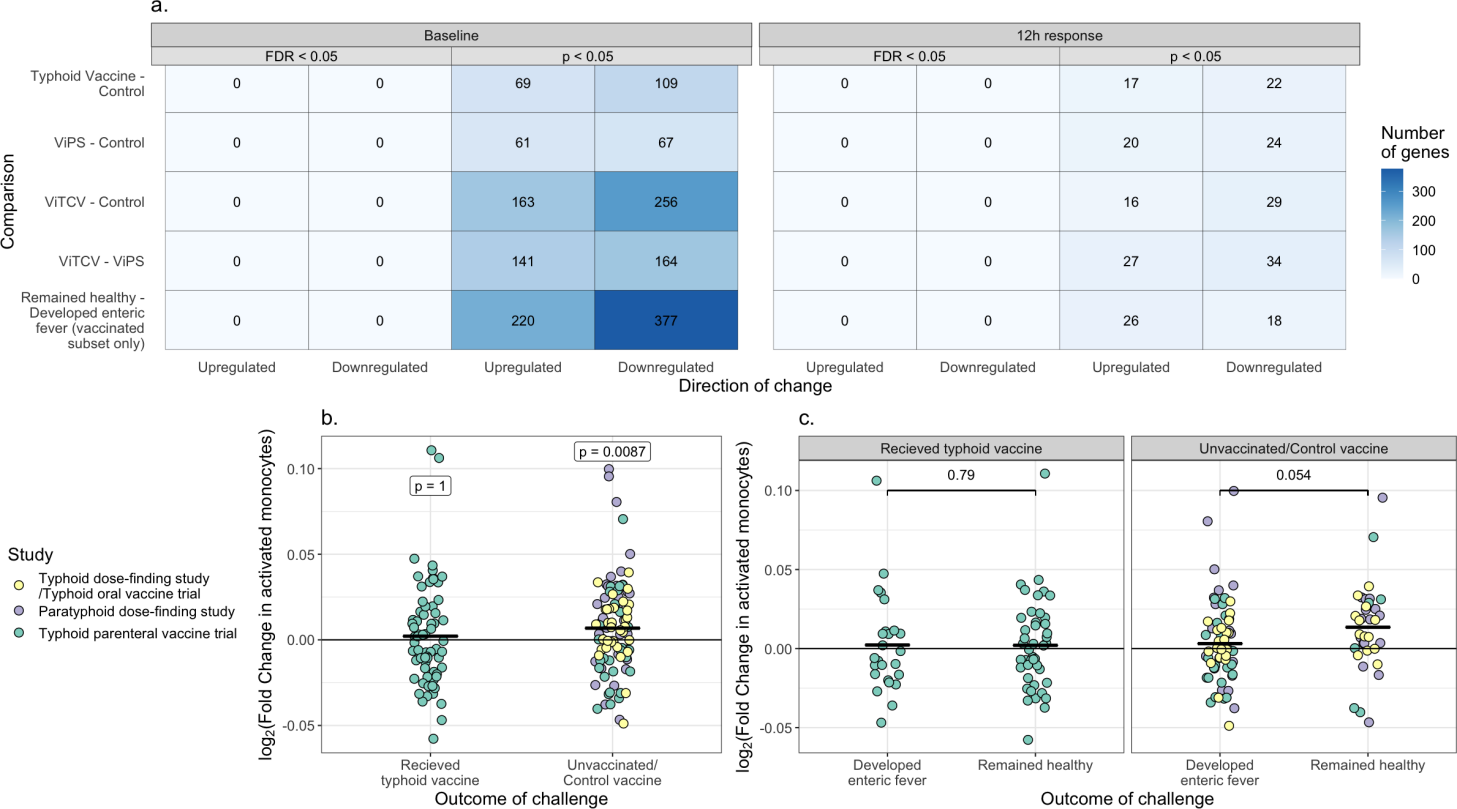
(**a**) Number of differentially expressed genes for each comparison between vaccine groups in the typhoid parenteral vaccine study. Numbers both before (*P* < 0.05) and after adjustment for multiple testing (FDR < 0.05) are shown. (**b**) Log_2_(fold change) in activated monocytes 12 hours post-challenge relative to baseline, grouped by vaccination status. White labels indicate the *P* values for a paired Wilcoxon-signed rank test comparing the 12 hours timepoint with baseline. Each point represents one participant, colored by study. The mean log_2_(fold change) is indicated by a black bar. (**c**) Log_2_(fold change) in activated monocytes 12 hours post-challenge relative to baseline, grouped by outcome of challenge and vaccination status. The *P* value for a Mann-Whitney test comparing the log_2_(fold changes) between groups is shown. Each point represents one participant, colored by study. The mean log_2_(fold change) is indicated by a black bar.

Finally, we investigated whether there were any transcriptional differences at baseline or in the 12 hours response between vaccine groups in the typhoid parenteral vaccine study. Both circadian and non-circadian genes were analyzed, as with the analysis of different outcome groups above. No genes were significant after adjusting for multiple testing (Fig. 6a).

Dividing challenge participants across all studies into vaccinated and unvaccinated/control groups, there was a significant rise in the predicted levels of activated monocytes 12 hours post-challenge in unvaccinated participants but not in vaccinated participants (*P* = 0.0087 and 1 respectively, paired Wilcoxon test, Fig. 6b). Furthermore, in the unvaccinated challenge participants, there was a trend toward greater activated monocytes 12 hours post-challenge in those who remained healthy relative compared with those who developed enteric fever (*P* = 0.054, logistic regression adjusting for study as a batch effect) which was not seen in vaccinated participants (*P* = 0.79, Fig. 6c).

## DISCUSSION

This is the first study to profile changes in the human blood transcriptome 12 hours after exposure to *S*. Typhi and *S*. Paratyphi A. 199 genes were transcriptionally upregulated 12 hours after challenge, suggesting a host response to exposure even at this early stage of infection. There was also an increase in plasma TNF-α and estimated activated monocytes 12 hours post-challenge. No differences in gene expression were identified between those who did and those who did not go on to develop enteric fever, indicating that this early host response occurs even in those who remain healthy despite exposure.

Specific genes upregulated 12 hours post-challenge included caspase *CASP4*, which plays a key role in inflammasome-mediated immunity to *Salmonella* infections (22, 23); *IFITM2*, which can inhibit intracellular bacterial growth in monocytes (24); and *RBCK1,* a component of a complex regulating NF-kB pathway activation (25). The kinetics of these changes, returning to baseline by 24 hours post-challenge, are consistent with data from historical challenge studies: participants treated with chloramphenicol 24 hours after challenge still developed blood-culture positive enteric fever, suggesting that by this time *S*. Typhi has reached an intracellular environment (26).

As transcriptional profiling was carried out on whole blood, changes in gene expression were driven not only by transcriptional regulation but also by changes in the proportions of different immune cells. Previously, single-cell RNA-sequencing has been used to characterize cell-specific responses to *ex vivo* infection with the related serovar *S*. Typhimurium (21). Exposed monocytes developed a distinct gene expression profile with 90% containing intracellular bacteria. We applied the resultant deconvolution algorithm to our data and found a small rise in monocytes estimated to be in this *Salmonella*-exposed activated state. However, as the rise was very small, and cell expression profiles were determined using a different serovar and *ex vivo*, this observation needs to be corroborated by future experimental data such as flow cytometry or single cell-RNA-sequencing. Nonetheless, it does support murine data suggesting that infected monocytes are responsible for both systemic *S*. Typhi dissemination and early innate responses to infection (6, 27). Furthermore, human data suggest *S*. Typhi interacts with and is found within monocytes at later stages of infection (14, 28). These results, together with previous research describing a rise in the ability of monocytes to bind *S*. Typhi 24 hours after challenge (29), could imply that monocytes are also involved in early human-*S*. Typhi interactions. The rise in the predicted number of activated monocytes was only marked for unvaccinated participants who remained healthy. If confirmed, this could suggest the early response may be protective, and that an alternative antibody-mediated mechanism of protection may take place in vaccinated participants; for example, antibody-dependent phagocytosis and killing (30) in the lamina propria.

In addition, we observed a rise in TNF-α 12 hours post-challenge in samples from a typhoid toxin human challenge study. A similar but non-significant rise has previously been observed in the typhoid dose-finding study [1.11-fold in the typhoid dose finding study (*P* = 0.07) (14) compared with 1.17-fold here in the typhoid toxin study (*P* = 0.01)]. We found that following *in vitro* stimulation with *S*. Typhi, TNF-α was predominantly produced by monocytes. However, further *ex vivo* experiments will be required to ascertain whether TNF-α is primarily expressed by monocytes or other cell types 12 hours post-challenge *in vivo*. One possibility is secretion of cytokines from the intestinal mucosa into the blood. However, organotypic models have found TNF-α to be primarily released by monocyte/macrophages rather than lymphocytes, fibroblasts, endothelial cells, or epithelial cells following *S*. Typhi or *S*. Paratyphi A stimulation (31, 32). Furthermore, *ex vivo* stimulation of intestinal biopsies with *S*. Typhi only elicited a non-significant increase in TNF-α (33). Nonetheless, *in vivo,* a number of cell types could be responsible for secretion, including CD8 T cells (34) and mucosal-associated invariant T cells (35).

Those who did not go on to develop enteric fever had broadly similar transcriptional responses to those who did in the hours after challenge. This implies that at a dose giving a 60%–75% attack rate, much of the protection against enteric fever may be mediated at a later stage of infection, although it is unclear if the change in the peripheral blood transcriptome reflects a mucosal or systemic host-pathogen interaction. While difficult to study in humans, mouse enteric fever models using *S*. Typhimurium as a challenge agent have previously characterized variation in host responses. One possibility is that differences in susceptibility are due to innate genetic variation. In 32 genetically distinct mouse strains orally challenged with *S*. Typhimurium, bacteria reached the spleen and liver of all strains but with considerable variation in bacterial load and survival after 7 days (36). Another study found that whereas three mouse strains had similar bacterial loads in the liver/spleen at day 1 after intravenous infection, there was significant variation by day 3 (37). Variation in pre-existing immunity may also exert an effect later in infection. For example, vaccinated and naïve mice had similar liver/spleen bacterial loads at day 1 after oral *S*. Typhimurium challenge but diverged by day 5 (38). Conversely, in another study where challenge was intravenously administered at a much lower dose, differences in liver/spleen load were already evident between vaccinated and naïve mice within 6 hours (39).

Here, we carried out transcriptional profiling of the bulk peripheral blood transcriptome. This allowed us to include changes in granulocyte gene expression and reduced the effect of blood processing on gene expression as samples were immediately lysed by a stabilizing reagent. However, this approach prevented us from finding how early enteric fever challenge affects individual cell populations. Detailed longitudinal and single-cell transcriptional profiling of the incubation period, with participants challenged at varying times of day, would be hugely valuable in elucidating mechanisms of early immunity and disease pathogenesis. This study was also limited to peripheral blood due to the invasiveness of gut, spleen, liver, or bone marrow biopsies. It may be that analyzing host responses in these tissues would be necessary to identify the timepoint at which those who develop enteric fever diverge from those who do not.

Together, our results indicate an early innate response to enteric fever 12 hours following challenge. Monocytes may play a key role in early innate responses to enteric fever. However, early responses were broadly similar between those who remained healthy and those who developed enteric fever, as well as between vaccination groups. A possible explanation is that protection is mediated at a later stage of infection, following systemic dissemination. Further investigation of the mechanism of protection, and its implications for effective vaccine design, is warranted.

## MATERIALS AND METHODS

### Human challenge studies

Samples were collected from six enteric fever human challenge studies (Table 1). Whereas, the typhoid- (15) and paratyphoid-dose finding (16) studies aimed to find the inoculum dose that would give an attack rate of 60%–75%, the typhoid oral vaccine trial (7), typhoid parenteral vaccine trial (17), typhoid toxin study (18), and re-challenge study (19) aimed to find the effect of oral vaccination, parenteral vaccination, typhoid toxin knockout, and prior challenge on attack rate, respectively. All studies were approved by the South Central-Oxford A Research Ethics Committee (10 /H0604/53, 11/SC/0302, 14/SC/0004, 14/SC/1427, 16/SC/0358, and 14/SC/1204), and all participants provided written informed consent. In the typhoid oral and parenteral vaccine trials, participants received a typhoid vaccine or placebo/control vaccine 28 days prior to challenge. Participants were challenged with *S*. Typhi Quailes strain, toxin-deficient *S*. Typhi SB6000 strain, or *S*. Paratyphi A NVGH308 strain suspended in sodium bicarbonate. Unchallenged control participants were given sodium bicarbonate alone. Enteric fever was defined as either an oral temperature ≥38°C sustained for ≥12 hours, or a positive blood culture. Participants were treated with ciprofloxacin or azithromycin following diagnosis or at 14 days if they did not develop enteric fever.

### Transcriptional profiling

Three milliliters of peripheral blood was collected in Tempus Blood RNA tubes before challenge (approximately 6–9 am) and approximately 12 and 24 hours following challenge. Total RNA was extracted using the Tempus Spin RNA Isolation Kit (Life Technologies).

RNA samples from individuals in the typhoid dose-finding study and typhoid oral vaccine trial were hybridized onto Illumina HT-12v4 bead-arrays at the Wellcome Trust Sanger Institute and Wellcome Trust Centre for Human Genetics, and fluorescent probe intensities captured with GenomeStudio software. Samples were hybridized in three batches. These two studies were grouped together for all following analyses. RNA samples from individuals in the paratyphoid dose-finding study and typhoid parenteral vaccine trial were poly-A selected (but not globin-depleted) and sequenced using a HiSeq V4 at the Wellcome Trust Sanger Institute. Fastq files from the same sample were concatenated, and prealignment quality control carried out using FASTQC. As all files had consistently high phred quality scores (>25) across their length, all were aligned to the human genome (GRCh38 Gencode version 26) using STAR-2.6.1c (40). Total reads per sample ranged from 16 to 44 million. Reads per gene were counted using the STAR GeneCounts mode.

### Publicly available circadian data sets

Two publicly available whole blood transcriptome data sets were downloaded to identify differential gene expression attributable to circadian rhythms: GSE48113 (12), profiled using an Agilent Whole Human Genome 4 × 44K custom oligonucleotide microarray, and GSM3122578, profiled by RNA-sequencing (13). Samples from GSM3122578 were aligned to the human genome (GRCh38 Gencode version 26) using STAR-2.6.1c and counted using STAR GeneCounts mode as above. As baseline and 24 hours post-challenge samples were taken at 6–9 am in the typhoid study and 12 hours post-challenge samples at 6–9 pm, data sets were subset to in-phase samples collected at these time points. Two samples from GSM3122578 were excluded on the basis of no mapped reads. Twenty morning samples and thirty-two evening samples were analyzed from GSE48113, and ten morning samples and nine evening samples from GSM3122578.

### Data processing

Microarray data sets, including GSE48113, were background corrected and quantile normalized, then the package lumi used to identify outliers for exclusion. On this basis, two samples from one of the typhoid dose-finding/typhoid oral vaccine trial batches were excluded from the raw data, which were then re-normalized. Probes from each array were aligned to the human genome assembly (GRCh38 GenCode version 26) using STAR-2.6.1c (40). Probes which did not align to genes were excluded, as were probes aligning to multiple genes. Where multiple probes corresponded to one gene symbol, the collapseRows function from the WGCNA R package was used to select the probe with the highest average mean as being representative, on the basis of this being the most reproducible approach (41). Genes corresponding to probes which were not significantly expressed over the negative controls (*P* > 0.05) in more than 37 samples (the number of baseline samples) in the typhoid dose-finding study/typhoid oral vaccine trial data set were excluded.

For the paratyphoid dose-finding study and typhoid parenteral vaccine trial RNA-sequencing data sets, principal component analysis was used for outlier detection, with no samples excluded on this basis. Non-protein coding and hemoglobin subunit genes were excluded. Count tables were filtered to exclude genes with <1 cpm in ≥31 samples (the number of baseline samples in control participants challenged with *S*. Typhi) and normalized using weighted trimmed mean of M-values scaling (edgeR).

### Differential gene expression analysis

For microarray data, a linear regression model was fitted using the limma package. Challenge dose and batch were both incorporated into the model as categorical covariates, where those in the typhoid oral vaccine trial and escalated dose group in the typhoid dose-finding study [10–50 × 10^3^ colony forming units (CFU)] were considered “high dose,” and those in the lower dose group (1–5 × 10^3^ CFU) in the typhoid dose-finding study were considered “low dose.” Analysis was paired by using participant ID as a blocking variable. For RNA-sequencing data, the count matrix was first transformed using limma voom. A linear regression model was fitted with vaccination status, sequence pool, and dose as categorical covariates, where the deescalated dose group (0.5–1 × 10^3^ CFU) in the paratyphoid challenge study was considered “low dose” and the higher dose group (1–5 × 10^3^) as “high dose.” Analysis was paired using participant ID as a blocking variable.

### Over-representation of genes relating to circadian rhythms

The results of differential expression analysis using limma were used to classify genes into differentially expressed after challenge (adjusted *P* value < 0.05) and not differentially expressed after challenge (adjusted *P* value > 0.05). Genes were also classified as potentially circadian (unadjusted *P* < 0.05 or absolute log_2_(fold change) >0.1 in the two circadian data sets) or not circadian. A contingency table, limited to genes present in both the enteric challenge and circadian data sets, was created. A fisher test was then used to test for over-representation.

### Transcriptional changes 12 hours post-challenge

To stringently exclude any genes that may be differentially expressed due to delayed processing or circadian rhythms, genes with an unadjusted *P* value < 0.05 or log_2_(fold change) >0.1 in either GSE48113 or GSM3122578 were excluded from analysis. The package MetaVolcanoR was then used to combine the differential expression results from the typhoid dose-finding/typhoid oral vaccine trial, typhoid parenteral vaccine trial, and paratyphoid dose-finding study using a random effects model.

The gene list generated by the differential gene expression meta-analysis was ranked by fold change, such that the most upregulated genes were at the top of the list and the most downregulated at the bottom. The gene lists were then input into a gene set enrichment analysis algorithm (42), in order to determine whether members of different blood transcriptional modules [groups of genes related by biological function or cell-specific expression (43)], were unevenly distributed in the gene list.

### Differential expression between subgroups

Genes that may be differentially expressed due to circadian rhythms were not excluded, on the basis that samples from different groups were subject to the same confounding factors. For different outcome groups, differences at baseline (Remained healthy baseline samples—Developed enteric fever baseline samples) and in response to challenge [(Remained healthy 12 hours post-challenge samples—Remained healthy baseline samples) − (Developed enteric fever 12 hours post-challenge samples – Developed Enteric Fever baseline samples)] were assessed by differential expression in limma. As above, the package MetaVolcanoR was then used to combine the differential expression results from the typhoid dose-finding/typhoid oral vaccine trial, typhoid parenteral vaccine trial, and paratyphoid dose-finding study using a random effects model.

For different dose groups, differences in response to challenge [(Higher dose 12 hours post-challenge samples – Higher dose baseline samples) – (Lower dose 12 hours post-challenge samples – Lower dose baseline samples)] were assessed by differential expression in limma for the typhoid dose-finding/typhoid oral vaccine trial and paratyphoid dose-finding study. Analysis of differences between vaccination groups was restricted to the typhoid parenteral vaccine trial, assessing differences at baseline, in the 12 hours response, and between outcome groups for vaccinated participants only.

### Deconvolution of the transcriptional response

RNA-sequencing data sets were normalized by library size to give counts per million, then log-transformed. CIBERSORT was used to estimate changes in immune cell populations *in silico*. Estimated cell fractions were compared with differential white blood cell counts taken at baseline in the typhoid dose-finding study and typhoid oral vaccine trial and absolute blood counts in the paratyphoid dose-finding study. Processed gene expression matrices were imported into MATLAB, and the dynamic deconvolution algorithm from https://github.com/noabossel/Dynamic-deconvolution-algorithm applied to estimate changes in monocytes in an activated infection-induced state. A paired Wilcoxon test was used to assess whether monocytes in an infection-induced state were significantly changed from baseline. Comparing those who developed enteric fever with those who did not at baseline, samples were unpaired with systematic differences in levels of predicted infection-induced state monocytes between studies. Therefore, a logistic regression model with study as a covariate was used. A Mann-Whitney test was used to assess whether there was a difference in the log_2_(fold change) in cells 12 hours post-challenge relative to baseline among different subgroups.

### Plasma cytokine quantification

Heparinized blood samples were collected and centrifuged at 3,000 rpm for 10 minutes at 4°C. The supernatant was transferred to a fresh tube before centrifuging a second time. The supernatant was then aliquoted and protease inhibitor added in a 1:40 dilution before storage at −80°C. Plasma aliquots at baseline and 12 hours post-challenge or mock challenge were assayed in duplicate using a MILLIPLEX MAP Kit with nine analytes (EGF, TGF-α, CXCL1, CD40L, IL1RA, IL2, IL6, IL8, and TNF-α) following manufacturer instructions. For values with fluorescence intensities falling below that of the lowest standard (3.2 pg/mL), the concentration was set to half that of the lowest standard (1.6 pg/mL). Duplicates were averaged.

### *In vitro* stimulation with *S*. typhi

To characterize cytokine secretion by different leukocyte subsets, 15 mL blood was collected from each of three healthy donors in a BD ethylenediaminetetraacetic acid vacutainer and layered onto an equal volume of Polymorphprep (Alere Technologies) then centrifuged at 500× g for 35 minutes at room temperature with the brake off. The granulocyte and peripheral blood mononuclear cells (PBMC) bands were transferred to separate Falcon tubes and topped up to 50 mL with phosphate-buffered saline (PBS). Cells were centrifuged at 250× g for 10 minutes then resuspended in 5 mL red blood cell lysis solution (eBioscience) for 5 minutes. The tube was once more topped up to 50 mL with PBS, centrifuged at 250 × g for 10 minutes, and the pellets resuspended in 4 mL PBS for cell counting. CD14 microbeads (Miltenyi Biotec) were diluted one in five in autoMACs running buffer and PBMCs suspended in 300 µL diluted beads. After 15 minutes at 4°C, 6 mL autoMACS running buffer was added, and the PBMCs centrifuged for 10 minutes at 250 G. PBMCs were then resuspended in 500 µL running buffer, and the CD14^+^ fraction (monocytes) and CD14^–^ fraction (lymphocytes) separated using an autoMACS Pro Separator. Viability was assessed using trypan blue and was high. Monocytes, lymphocytes, and granulocytes were counted using a hemocytometer, suspended in Roswell Park Memorial Institute (RPMI) 1,640 Medium and seeded in duplicate (2 wells per donor per cell type) in a 96 wells plate at a density of 1,00,000 cells per well. Frozen stocks of 10^7^ CFU/ml Quailes strain *S*. Typhi were thawed and washed twice with RPMI. Cells were inoculated with *S*. Typhi at a multiplicity of infection of 10 or RPMI as a negative control. After 50 minutes, gentamycin was added to a final concentration of 200 µg/mL. Four hours post-inoculation, the plate was centrifuged at 400× g for 4 minutes, and the supernatants filtered using 0.22 µm cellulose acetate Spin-X centrifuge tube filters. Samples were assayed in duplicate using a MILLIPLEX MAP kit with nine analytes (EGF, TGF-α, CXCL1, CD40L, IL1RA, IL2, IL6, IL8, and TNF-α). For values with fluorescence intensities falling below that of the lowest standard (3.2 pg/mL), the concentration was set to half that of the lowest standard (1.6 pg/mL). Duplicates were averaged.

## Data Availability

The transcriptional data produced by this study have been deposited in the Gene Expression Omnibus (GEO) database under GSE225930 (typhoid dose-finding study and typhoid oral vaccine trial), GSE114033 (paratyphoid dose-finding study, baseline), GSE224006 (paratyphoid dose-finding study, 12 hours post-challenge) and GSE217667 (typhoid parenteral vaccine trial).

## SUPPLEMENTAL MATERIAL

Supplemental Document 1 iai.00108-23-s0001.xlsx Differential gene expression results for each individual dataset.

Supplemental Document 2 iai.00108-23-s0002.xlsx Differential gene expression results for combined datasets (shared non-circadian genes only).

Supplemental figures and tables iai.00108-23-s0003.docx Figures S1 to S4 and Table S1.

ASM does not own the copyrights to Supplemental Material that may be linked to, or accessed through, an article. The authors have granted ASM a non-exclusive, world-wide license to publish the Supplemental Material files. Please contact the corresponding author directly for reuse.

## References

[1] Stanaway JD , Reiner RC , Blacker BF , Goldberg EM , Khalil IA , Troeger CE , Andrews JR , Bhutta ZA , Crump JA , Im J , Marks F , Mintz E , Park SE , Zaidi AKM , Abebe Z , Abejie AN , Adedeji IA , Ali BA , Amare AT , Atalay HT , Avokpaho E , Bacha U , Barac A , Bedi N , Berhane A , Browne AJ , Chirinos JL , Chitheer A , Dolecek C , El Sayed Zaki M , Eshrati B , Foreman KJ , Gemechu A , Gupta R , Hailu GB , Henok A , Hibstu DT , Hoang CL , Ilesanmi OS , Iyer VJ , Kahsay A , Kasaeian A , Kassa TD , Khan EA , Khang Y-H , Magdy Abd El Razek H , Melku M , Mengistu DT , Mohammad KA , Mohammed S , Mokdad AH , Nachega JB , Naheed A , Nguyen CT , Nguyen HLT , Nguyen LH , Nguyen NB , Nguyen TH , Nirayo YL , Pangestu T , Patton GC , Qorbani M , Rai RK , Rana SM , Ranabhat CL , Roba KT , Roberts NLS , Rubino S , Safiri S , Sartorius B , Sawhney M , Shiferaw MS , Smith DL , Sykes BL , Tran BX , Tran TT , Ukwaja KN , Vu GT , Vu LG , Weldegebreal F , Yenit MK , Murray CJL , Hay SI . 2019. The global burden of typhoid and paratyphoid fevers: a systematic analysis for the global burden of disease study 2017. Lancet Infect Dis 19:369–381. doi:10.1016/S1473-3099(18)30685-6 30792131 PMC6437314

[2] Shakya M , Colin-Jones R , Theiss-Nyland K , Voysey M , Pant D , Smith N , Liu X , Tonks S , Mazur O , Farooq YG , Clarke J , Hill J , Adhikari A , Dongol S , Karkey A , Bajracharya B , Kelly S , Gurung M , Baker S , Neuzil KM , Shrestha S , Basnyat B , Pollard AJ , TyVAC Nepal Study Team . 2019. Phase 3 efficacy analysis of a typhoid conjugate vaccine trial in Nepal. N Engl J Med 381:2209–2218. doi:10.1056/NEJMoa1905047 31800986 PMC6785806

[3] Patel PD , Patel P , Liang Y , Meiring JE , Misiri T , Mwakiseghile F , Tracy JK , Masesa C , Msuku H , Banda D , Mbewe M , Henrion M , Adetunji F , Simiyu K , Rotrosen E , Birkhold M , Nampota N , Nyirenda OM , Kotloff K , Gmeiner M , Dube Q , Kawalazira G , Laurens MB , Heyderman RS , Gordon MA , Neuzil KM , TyVAC Malawi Team . 2021. Safety and efficacy of a typhoid conjugate vaccine in malawian children. N Engl J Med 385:1104–1115. doi:10.1056/NEJMoa2035916 34525285 PMC8202713

[4] Snyder MJ , Hornick RB , McCrumb FR , Mors LJ , Woodward TE . 1963. Asymptomatic Typhoidal bacteremia in volunteers. Antimicrob Agents Chemother (Bethesda) 161:604–607. doi:10.1056/NEJM197009242831306 14274970

[5] Raffatellu M , Wilson RP , Winter SE , Bäumler AJ . 2008. Clinical pathogenesis of typhoid fever. J Infect Dev Ctries 2:260–266. doi:10.3855/jidc.219 19741286

[6] Dougan G , Baker S . 2014. Salmonella enterica serovar typhi and the pathogenesis of typhoid fever. Annu Rev Microbiol 68:317–336. doi:10.1146/annurev-micro-091313-103739 25208300

[7] Darton TC , Jones C , Blohmke CJ , Waddington CS , Zhou L , Peters A , Haworth K , Sie R , Green CA , Jeppesen CA , Moore M , Thompson BAV , John T , Kingsley RA , Yu L-M , Voysey M , Hindle Z , Lockhart S , Sztein MB , Dougan G , Angus B , Levine MM , Pollard AJ . 2016. Using a human challenge model of infection to measure vaccine efficacy: a randomised, controlled trial comparing the typhoid vaccines M01ZH09 with placebo and Ty21a. PLoS Negl Trop Dis 10:e0004926. doi:10.1371/journal.pntd.0004926 27533046 PMC4988630

[8] Darton TC , Zhou L , Blohmke CJ , Jones C , Waddington CS , Baker S , Pollard AJ . 2017. Blood culture-PCR to optimise typhoid fever diagnosis after controlled human infection identifies frequent asymptomatic cases and evidence of primary bacteraemia. J Infect 74:358–366. doi:10.1016/j.jinf.2017.01.006 28130144 PMC5345565

[9] Huang Y , Zaas AK , Rao A , Dobigeon N , Woolf PJ , Veldman T , Øien NC , McClain MT , Varkey JB , Nicholson B , Carin L , Kingsmore S , Woods CW , Ginsburg GS , Hero AO . 2011. Temporal dynamics of host molecular responses differentiate symptomatic and asymptomatic influenza a infection. PLoS Genet 7:e1002234. doi:10.1371/journal.pgen.1002234 21901105 PMC3161909

[10] Proud D , Turner RB , Winther B , Wiehler S , Tiesman JP , Reichling TD , Juhlin KD , Fulmer AW , Ho BY , Walanski AA , Poore CL , Mizoguchi H , Jump L , Moore ML , Zukowski CK , Clymer JW . 2008. Gene expression profiles during in vivo human rhinovirus infection: insights into the host response. Am J Respir Crit Care Med 178:962–968. doi:10.1164/rccm.200805-670OC 18658112

[11] Ockenhouse CF , Hu W , Kester KE , Cummings JF , Stewart A , Heppner DG , Jedlicka AE , Scott AL , Wolfe ND , Vahey M , Burke DS . 2006. Common and divergent immune response signaling pathways discovered in peripheral blood mononuclear cell gene expression patterns in presymptomatic and clinically apparent malaria. Infect Immun 74:5561–5573. doi:10.1128/IAI.00408-06 16988231 PMC1594921

[12] Archer SN , Laing EE , Möller-Levet CS , van der Veen DR , Bucca G , Lazar AS , Santhi N , Slak A , Kabiljo R , von Schantz M , Smith CP , Dijk D-J . 2014. Mistimed sleep disrupts circadian regulation of the human transcriptome. Proc Natl Acad Sci U S A 111:E682–91. doi:10.1073/pnas.1316335111 24449876 PMC3926083

[13] Braun R , Kath WL , Iwanaszko M , Kula-Eversole E , Abbott SM , Reid KJ , Zee PC , Allada R . 2018. Universal method for robust detection of circadian state from gene expression. Proc Natl Acad Sci U S A 115:E9247–E9256. doi:10.1073/pnas.1800314115 30201705 PMC6166804

[14] Blohmke CJ , Darton TC , Jones C , Suarez NM , Waddington CS , Angus B , Zhou L , Hill J , Clare S , Kane L , Mukhopadhyay S , Schreiber F , Duque-Correa MA , Wright JC , Roumeliotis TI , Yu L , Choudhary JS , Mejias A , Ramilo O , Shanyinde M , Sztein MB , Kingsley RA , Lockhart S , Levine MM , Lynn DJ , Dougan G , Pollard AJ . 2016. Interferon-driven alterations of the host's amino acid metabolism in the pathogenesis of typhoid fever. J Exp Med 213:1061–1077. doi:10.1084/jem.20151025 27217537 PMC4886356

[15] Waddington CS , Darton TC , Jones C , Haworth K , Peters A , John T , Thompson BAV , Kerridge SA , Kingsley RA , Zhou L , Holt KE , Yu L-M , Lockhart S , Farrar JJ , Sztein MB , Dougan G , Angus B , Levine MM , Pollard AJ . 2014. An outpatient, ambulant-design, controlled human infection model using escalating doses of Salmonella typhi challenge delivered in sodium bicarbonate solution. Clin Infect Dis 58:1230–1240. doi:10.1093/cid/ciu078 24519873 PMC3982839

[16] Dobinson HC , Gibani MM , Jones C , Thomaides-Brears HB , Voysey M , Darton TC , Waddington CS , Campbell D , Milligan I , Zhou L , Shrestha S , Kerridge SA , Peters A , Stevens Z , Podda A , Martin LB , D’Alessio F , Thanh DP , Basnyat B , Baker S , Angus B , Levine MM , Blohmke CJ , Pollard AJ . 2017. Evaluation of the clinical and microbiological response to Salmonella paratyphi a infection in the first paratyphoid human challenge model. Clin Infect Dis 64:1066–1073. doi:10.1093/cid/cix042 28158395 PMC5439345

[17] Jin C , Gibani MM , Moore M , Juel HB , Jones E , Meiring J , Harris V , Gardner J , Nebykova A , Kerridge SA , Hill J , Thomaides-Brears H , Blohmke CJ , Yu L-M , Angus B , Pollard AJ . 2017. Efficacy and immunogenicity of a VI-tetanus toxoid conjugate vaccine in the prevention of typhoid fever using a controlled human infection model of salmonella typhi: a randomised controlled, phase 2B trial. Lancet 390:2472–2480. doi:10.1016/S0140-6736(17)32149-9 28965718 PMC5720597

[18] Gibani MM , Jones E , Barton A , Jin C , Meek J , Camara S , Galal U , Heinz E , Rosenberg-Hasson Y , Obermoser G , Jones C , Campbell D , Black C , Thomaides-Brears H , Darlow C , Dold C , Silva-Reyes L , Blackwell L , Lara-Tejero M , Jiao X , Stack G , Blohmke CJ , Hill J , Angus B , Dougan G , Galán J , Pollard AJ . 2019. Investigation of the role of typhoid toxin in acute typhoid fever in a human challenge model. Nat Med 25:1082–1088. doi:10.1038/s41591-019-0505-4 31270506 PMC6892374

[19] Gibani MM , Jin C , Shrestha S , Moore M , Norman L , Voysey M , Jones E , Blackwell L , Thomaides-Brears H , Hill J , Blohmke CJ , Dobinson HC , Baker P , Jones C , Campbell D , Mujadidi YF , Plested E , Preciado-Llanes L , Napolitani G , Simmons A , Gordon MA , Angus B , Darton TC , Cerundulo V , Pollard AJ . 2020. Homologous and heterologous re-challenge with Salmonella typhi and salmonella paratyphi a in a randomised controlled human infection model. PLoS Negl Trop Dis 14:e0008783. doi:10.1371/journal.pntd.0008783 33079959 PMC7598925

[20] Prada C , Lima D , Nakaya H . 2019. MetaVolcanoR: gene expression meta-analysis visualization tool

[21] Bossel Ben-Moshe N , Hen-Avivi S , Levitin N , Yehezkel D , Oosting M , Joosten LAB , Netea MG , Avraham R . 2019. Predicting bacterial infection outcomes using single cell RNA-sequencing analysis of human immune cells. Nat Commun 10:3266. doi:10.1038/s41467-019-11257-y 31332193 PMC6646406

[22] Knodler LA , Crowley SM , Sham HP , Yang H , Wrande M , Ma C , Ernst RK , Steele-Mortimer O , Celli J , Vallance BA . 2014. Noncanonical inflammasome activation of caspase-4/caspase-11 mediates epithelial defenses against enteric bacterial pathogens. Cell Host Microbe 16:249–256. doi:10.1016/j.chom.2014.07.002 25121752 PMC4157630

[23] Mylona E , Sanchez-Garrido J , Hoang Thu TN , Dongol S , Karkey A , Baker S , Shenoy AR , Frankel G . 2021. Very long o-antigen chains of Salmonella paratyphi a inhibit inflammasome activation and pyroptotic cell death. Cell Microbiol 23:e13306. doi:10.1111/cmi.13306 33355403 PMC8609438

[24] Ranjbar S , Haridas V , Jasenosky LD , Falvo JV , Goldfeld AE . 2015. A role for IFITM proteins in restriction of mycobacterium tuberculosis infection. Cell Rep 13:874–883. doi:10.1016/j.celrep.2015.09.048 26565900 PMC4916766

[25] Boisson B , Laplantine E , Prando C , Giliani S , Israelsson E , Xu Z , Abhyankar A , Israël L , Trevejo-Nunez G , Bogunovic D , Cepika A-M , MacDuff D , Chrabieh M , Hubeau M , Bajolle F , Debré M , Mazzolari E , Vairo D , Agou F , Virgin HW , Bossuyt X , Rambaud C , Facchetti F , Bonnet D , Quartier P , Fournet J-C , Pascual V , Chaussabel D , Notarangelo LD , Puel A , Israël A , Casanova J-L , Picard C . 2012. Immunodeficiency, autoinflammation and amylopectinosis in humans with inherited HOIL-1 and LUBAC deficiency. Nat Immunol 13:1178–1186. doi:10.1038/ni.2457 23104095 PMC3514453

[26] Hornick RB , Greisman SE , Woodward TE , DuPont HL , Dawkins AT , Snyder MJ . 1970. Typhoid fever: pathogenesis and immunologic control. 2. N Engl J Med 283:739–746. doi:10.1056/NEJM197010012831406 4916916

[27] Tam MA , Rydström A , Sundquist M , Wick MJ . 2008. Early cellular responses to Salmonella infection: dendritic cells, monocytes, and more. Immunol Rev 225:140–162. doi:10.1111/j.1600-065X.2008.00679.x 18837781

[28] Wain J , Diep TS , Ho VA , Walsh AM , Nguyen TT , Parry CM , White NJ . 1998. Quantitation of bacteria in blood of typhoid fever patients and relationship between counts and clinical features, transmissibility, and antibiotic resistance. J Clin Microbiol 36:1683–1687. doi:10.1128/JCM.36.6.1683-1687.1998 9620400 PMC104900

[29] Toapanta FR , Bernal PJ , Fresnay S , Darton TC , Jones C , Waddington CS , Blohmke CJ , Dougan G , Angus B , Levine MM , Pollard AJ , Sztein MB . 2015. Oral wild-type Salmonella typhi challenge induces activation of circulating monocytes and dendritic cells in individuals who develop typhoid disease. PLoS Negl Trop Dis 9:e0003837. doi:10.1371/journal.pntd.0003837 26065687 PMC4465829

[30] Jin C , Hill J , Gunn BM , Yu W-H , Dahora LC , Jones E , Johnson M , Gibani MM , Spreng RL , Alam SM , Nebykova A , Juel HB , Dennison SM , Seaton KE , Fallon JK , Tomaras GD , Alter G , Pollard AJ . 2021. Vi-specific serological correlates of protection for typhoid fever. J Exp Med 218:e20201116. doi:10.1084/jem.20201116 33180929 PMC7668386

[31] Salerno-Gonçalves R , Galen JE , Levine MM , Fasano A , Sztein MB . 2018. Manipulation of Salmonella typhi gene expression impacts innate cell responses in the human intestinal mucosa. Front Immunol 9:2543. doi:10.3389/fimmu.2018.02543 30443257 PMC6221971

[32] Salerno-Goncalves R , Kayastha D , Fasano A , Levine MM , Sztein MB . 2019. Crosstalk between leukocytes triggers differential immune responses against Salmonella enterica serovars typhi and paratyphi. PLoS Negl Trop Dis 13:e0007650. doi:10.1371/journal.pntd.0007650 31412039 PMC6709971

[33] Nickerson KP , Senger S , Zhang Y , Lima R , Patel S , Ingano L , Flavahan WA , Kumar DKV , Fraser CM , Faherty CS , Sztein MB , Fiorentino M , Fasano A . 2018. Salmonella typhi colonization provokes extensive transcriptional changes aimed at evading host mucosal immune defense during early infection of human intestinal tissue. EBioMedicine 31:92–109. doi:10.1016/j.ebiom.2018.04.005 29735417 PMC6013756

[34] Fresnay S , McArthur MA , Magder LS , Darton TC , Jones C , Waddington CS , Blohmke CJ , Angus B , Levine MM , Pollard AJ , Sztein MB . 2017. Importance of salmonella typhi-responsive CD8+ T cell immunity in a human typhoid fever challenge model. Front Immunol 8:208. doi:10.3389/fimmu.2017.00208 28303138 PMC5332428

[35] Salerno-Gonçalves R , Fresnay S , Magder L , Darton TC , Waddington CS , Blohmke CJ , Angus B , Levine MM , Pollard AJ , Sztein MB . 2022. Mucosal-associated invariant T cells exhibit distinct functional signatures associated with protection against typhoid fever. Cell Immunol 378:104–572. doi:10.1016/j.cellimm.2022.104572 PMC9377420 35772315

[36] Scoggin K , Lynch R , Gupta J , Nagarajan A , Sheffield M , Elsaadi A , Bowden C , Aminian M , Peterson A , Adams LG , Kirby M , Threadgill DW , Andrews-Polymenis HL . 2022. Genetic background influences survival of infections with Salmonella enterica serovar typhimurium in the collaborative cross. PLoS Genet 18:e1010075. doi:10.1371/journal.pgen.1010075 35417454 PMC9067680

[37] Sebastiani G , Blais V , Sancho V , Vogel SN , Stevenson MM , Gros P , Lapointe J-M , Rivest S , Malo D . 2002. Host immune response to salmonella enterica serovar typhimurium infection in mice derived from wild strains. Infect Immun 70:1997–2009. doi:10.1128/IAI.70.4.1997-2009.2002 11895964 PMC127833

[38] Heithoff DM , Enioutina EY , Daynes RA , Sinsheimer RL , Low DA , Mahan MJ . 2001. Salmonella DNA adenine methylase mutants confer cross-protective immunity. Infect Immun 69:6725–6730. doi:10.1128/IAI.69.11.6725-6730.2001 11598044 PMC100049

[39] Coward C , Restif O , Dybowski R , Grant AJ , Maskell DJ , Mastroeni P . 2014. The effects of vaccination and immunity on bacterial infection dynamics in vivo. PLoS Pathog 10:e1004359. doi:10.1371/journal.ppat.1004359 25233077 PMC4169467

[40] Dobin A , Davis CA , Schlesinger F , Drenkow J , Zaleski C , Jha S , Batut P , Chaisson M , Gingeras TR . 2013. STAR: ultrafast universal RNA-seq aligner. Bioinformatics 29:15–21. doi:10.1093/bioinformatics/bts635 23104886 PMC3530905

[41] Miller JA , Cai C , Langfelder P , Geschwind DH , Kurian SM , Salomon DR , Horvath S . 2011. Strategies for aggregating gene expression data: the collapserows R function. BMC Bioinformatics 12:322. doi:10.1186/1471-2105-12-322 21816037 PMC3166942

[42] Subramanian A , Tamayo P , Mootha VK , Mukherjee S , Ebert BL , Gillette MA , Paulovich A , Pomeroy SL , Golub TR , Lander ES , Mesirov JP . 2005. Gene set enrichment analysis: a knowledge-based approach for interpreting genome-wide expression profiles. Proc Natl Acad Sci U S A 102:15545–15550. doi:10.1073/pnas.0506580102 16199517 PMC1239896

[43] Li S , Rouphael N , Duraisingham S , Romero-Steiner S , Presnell S , Davis C , Schmidt DS , Johnson SE , Milton A , Rajam G , Kasturi S , Carlone GM , Quinn C , Chaussabel D , Palucka AK , Mulligan MJ , Ahmed R , Stephens DS , Nakaya HI , Pulendran B . 2014. Molecular signatures of antibody responses derived from a systems biology study of five human vaccines. Nat Immunol 15:195–204. doi:10.1038/ni.2789 24336226 PMC3946932

