# Supplementary material for: Early transcriptional responses to human enteric fever challenge
Source: Infect Immun. 2023 Sep 19;91(10):e00108-23. doi: 10.1128/iai.00108-23 (PMC10581002; doi:10.1128/iai.00108-23)
Supplement: Supplemental figures and tables — Figures S1 to S4 and Table S1. [file iai.00108-23-s0003.docx]

**Supplementary Figures and Tables**


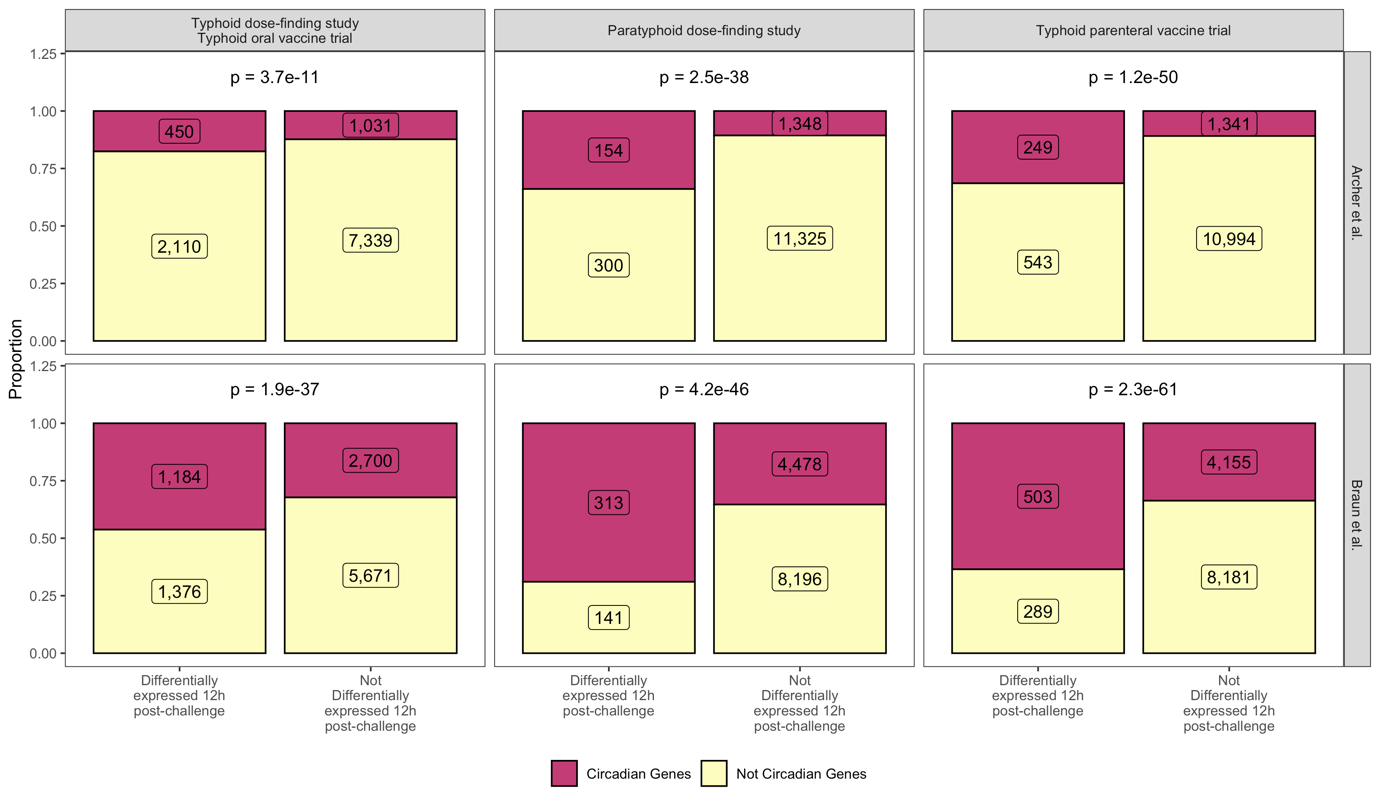


Figure S1: Proportion of circadian genes (p < 0.05 or absolute log_2_(fold change) > 0.1 for differential expression in the evening versus the morning) amongst genes differentially expressed following enteric fever challenge. Two datasets were used to identify putative circadian genes: Archer at al. (microarray) and Braun et al. (RNA-sequencing), The number of genes in each category is indicated. The proportion of circadian genes amongst genes not differentially expressed 12 hours post-enteric fever challenge is shown for comparison. P values for a fisher test are shown in each panel.


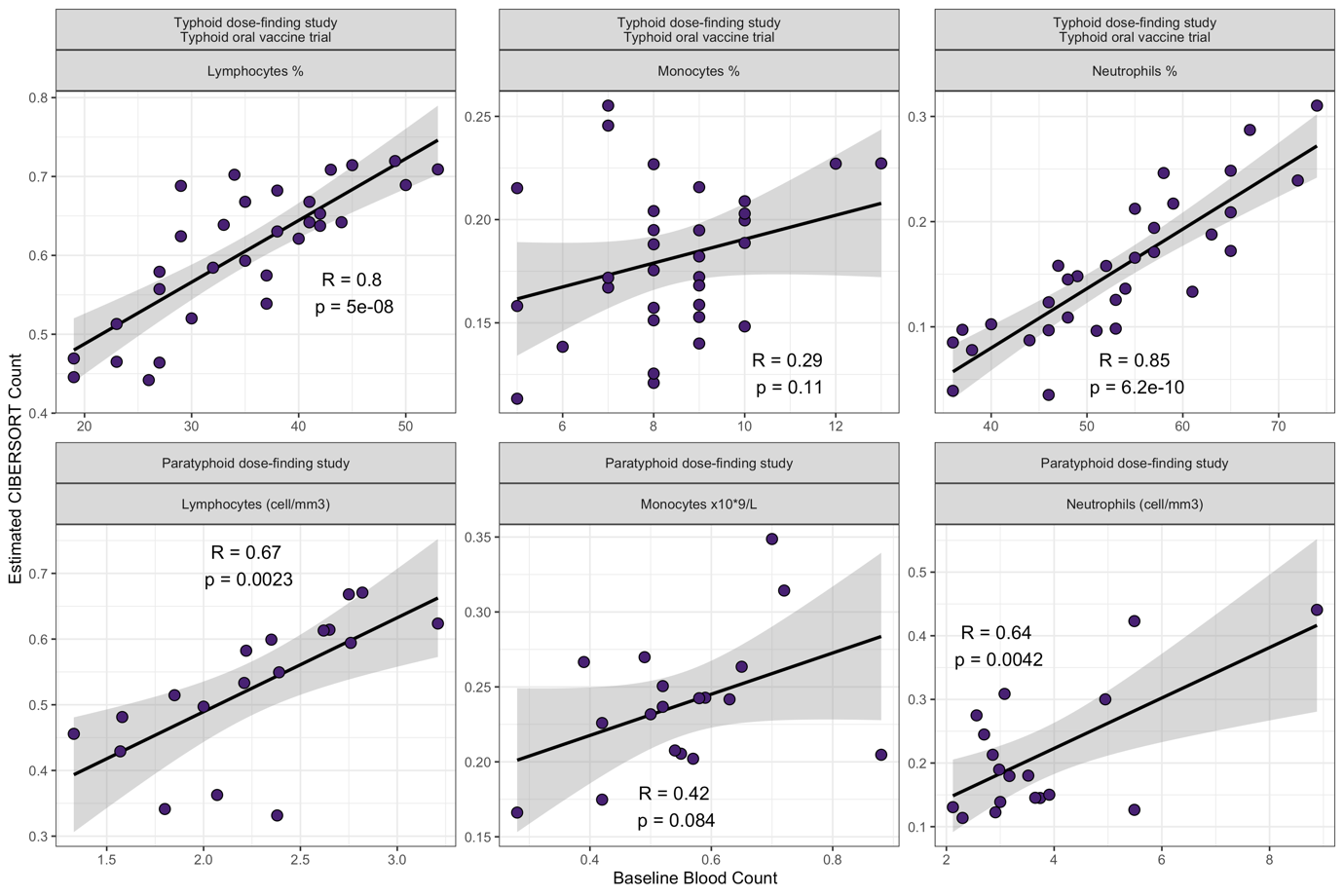


Figure S2: Correlation between baseline blood counts and the blood count estimated by CIBERSORT from bulk transcriptomics data. For the typhoid dose-finding and oral vaccine studies, differential blood counts were collected, whereas for the paratyphoid dose-finding study absolute blood counts were collected. Each point represents one participant. A linear line-of-best fit with shaded 95% confidence intervals is indicated, as is a Pearson's product moment correlation coefficient (R) and p value of a test for R being zero.


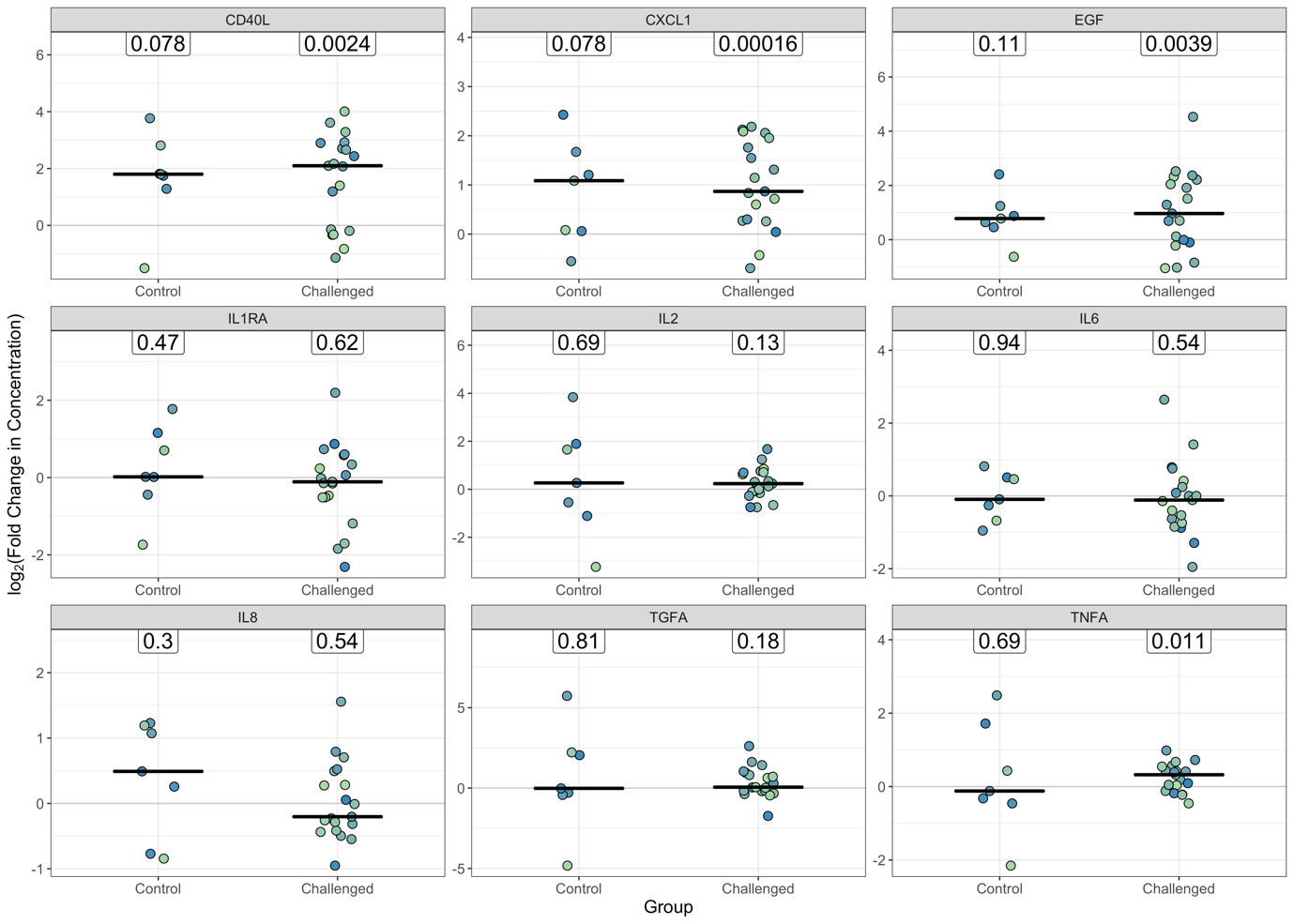


Figure S3: The log_2_(fold change) of cytokines 12 hours after baseline in unchallenged control participants and challenged participants. Each point represents one participant. For each cytokine and group the median is indicated by a horizontal line. White labels indicate the p values for a paired Wilcoxon-signed rank test comparing concentrations at the 12 hour timepoint with baseline.


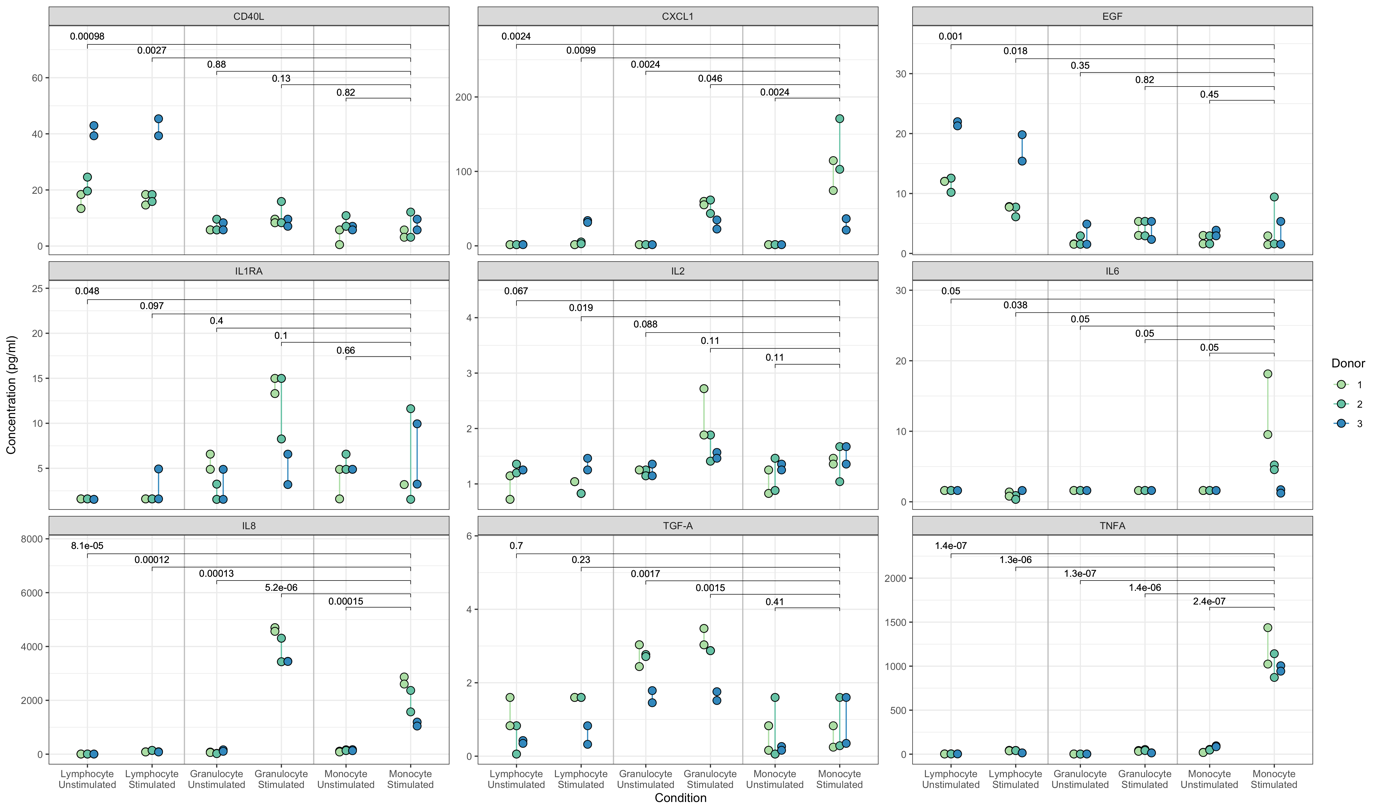


Figure S4: Cytokine production by granulocytes, monocytes and lymphocytes from three donors stimulated with *S*. Typhi at an MOI of 10 after 4 hours, relative to an unstimulated control. The p value for a linear mixed effects model with blood donor modelled as a batch is indicated. Points are coloured by donor.

Table S1: Non-circadian genes significantly different in the 12 hour response between dose groups (false discovery rate < 0.05) in the paratyphoid dose-finding study.

| Gene | High dose 12h response vs low dose group 12h response | | Log_2_(fold change) relative to baseline | |
| --- | --- | --- | --- | --- |
|  | P value | Adjusted p value | High dose | Low dose |
| ZNF207 | 0.000021 | 0.031 | 0.24 | -0.07 |
| ERO1B | 0.000036 | 0.039 | 0.32 | -0.10 |
| PRPF4B | 0.000004 | 0.026 | 0.19 | -0.07 |
| TIA1 | 0.000012 | 0.026 | 0.50 | -0.05 |
| RBM39 | 0.000008 | 0.026 | 0.32 | -0.04 |
| ZRANB2 | 0.000014 | 0.026 | 0.39 | -0.10 |
| TIAL1 | 0.000026 | 0.034 | 0.17 | -0.08 |
| TTC14 | 0.000007 | 0.026 | 0.53 | -0.08 |
| N4BP2L2 | 0.000012 | 0.026 | 0.36 | -0.08 |
| ZNF224 | 0.000049 | 0.047 | 0.27 | -0.16 |
